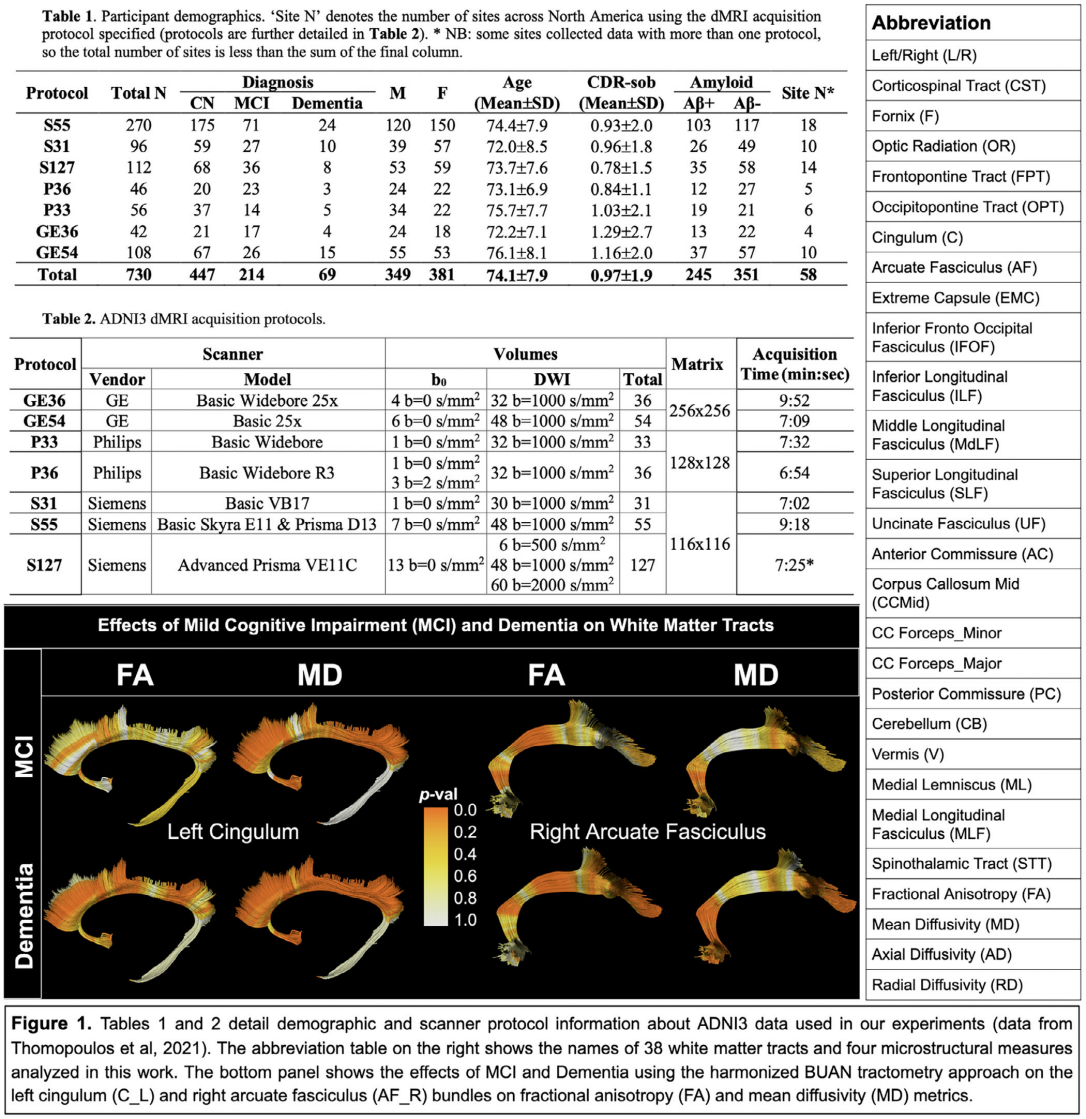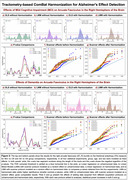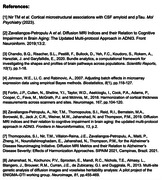# Harmonized Tractometry Visualizes Alzheimer’s Disease Effects on the Brain’s White Matter Tract Microstructure

**DOI:** 10.1002/alz.089548

**Published:** 2025-01-09

**Authors:** Bramsh Q Chandio, Julio E Villalon Reina, Talia M Nir, Sophia I Thomopoulos, Yixue Feng, Sebastian M Benavidez, Neda Jahanshad, Jaroslaw Harezlak, Eleftherios Garyfallidis, Paul M. Thompson

**Affiliations:** ^1^ Imaging Genetics Center, Mark and Mary Stevens Neuroimaging and Informatics Institute, Keck School of Medicine, University of Southern California, Marina del Rey, CA USA; ^2^ Imaging Genetics Center, Mark and Mary Stevens Neuroimaging & Informatics Institute, University of Southern California, Marina del Rey, CA USA; ^3^ Imaging Genetics Center, Mark and Mary Stevens Neuroimaging & Informatics Institute, Keck School of Medicine, University of Southern California, Marina del Rey, CA USA; ^4^ Indiana University School of Public Health‐ Bloomington, Bloomington, IN USA; ^5^ Indiana University Bloomington, Bloomington, IN USA

## Abstract

**Background:**

Diffusion MRI (dMRI) metrics of brain microstructure offer valuable insight into Alzheimer’s disease (AD) pathology; recent reports have identified dMRI metrics that (1) tightly link with CSF or PET measures of amyloid and tau burden; and (2) mediate the relationship between CSF markers of AD and delayed logical memory performance, commonly impaired in early AD [1,2].

To better localize white matter tract disruption in AD, our BUndle ANalytic (BUAN) [3] tractometry pipeline allows principled use of statistical methods to map factors affecting microstructural metrics along the 3D length of the brain’s fiber tracts. Here, we extended BUAN to pool data from multiple scanning protocols/sites ‐ using a new **harmonized tractometry** approach, based on ComBat [4,5], a widely‐used harmonization method modeling variations in multi‐site datasets due to site‐ and scanner‐specific effects.

**Method:**

We illustrate the effects of integrating ComBat into our BUAN tractometry pipeline. Analyzing Alzheimer’s Disease Neuroimaging Initiative (ADNI3) [6] data, we examined the impact of mild cognitive impairment (MCI) and AD on 38 white matter tracts, comparing results with and without harmonization.

We analyzed data from 730 ADNI3 participants, scanned with seven dMRI protocols. After data preprocessing with the ADNI3 protocol [7,8], BUAN extracted 38 bundles and created along‐tract bundle profiles for microstructural metrics: FA, MD, AD, and RD (see Figure 1 for full names). ComBat was applied to each point in bundle profiles to correct for scanner protocol effects and linear mixed models (LMM) were used to study the group differences in MCI and AD versus cognitively healthy controls (CN).

**Result:**

Harmonized BUAN maps reveal AD and MCI effects throughout specific tracts (e.g., the left cingulum and right arcuate fasciculus; Figure 1, *bottom panel*). ComBat harmonization enhanced the sensitivity to detect group differences compared to LMM without scanner correction. Adding data ‐ even from different scanner protocols ‐ boosted power (Figure 2f‐g).

**Conclusion:**

In this application of harmonization in neurodegenerative tractometry analysis, we integrated ComBat into BUAN tractometry to merge dMRI data from diverse scanning protocols and sites. Future research will examine various ComBat versions and deep‐learning approaches to track AD pathology effects on the brain’s neural circuitry.